# Induction of Immune Responses in Mice and Newborn Piglets by Oral Immunization with Recombinant *Lactococcus lactis* Expressing S1 and M Proteins of Porcine Epidemic Diarrhea Virus

**DOI:** 10.3390/microorganisms13040714

**Published:** 2025-03-21

**Authors:** Xiulei Cai, Zhikui Wang, Xinping Yan, Xu Wang, Xiaoxue Yue, Hongliang Zhang

**Affiliations:** College of Veterinary Medicine, Qingdao Agricultural University, Qingdao 266109, China; xlcai_99@163.com (X.C.); wangzhikui@stu.qau.edu.cn (Z.W.); 18315901099@163.com (X.Y.); wangxu1006@yeah.net (X.W.); yuexiaoxue2024@163.com (X.Y.)

**Keywords:** porcine epidemic diarrhea virus, *Lactococcus lactis*, oral immunization, immunogenicity evaluation, mice and piglets

## Abstract

Porcine epidemic diarrhea (PED) is a severe gastrointestinal disease caused by the porcine epidemic diarrhea virus (PEDV), a virus that spreads through the intestinal tract, leading to significant economic losses in the global swine industry. Therefore, compared to traditional injection method, developing vaccines that effectively stimulate the mucosal immune system to induce a protective immune response is crucial for PED prevention. This study evaluated the immunogenicity of recombinant *Lactococcus lactis* (*L. lactis*) strains expressing the PEDV S1 and M proteins (MG1363/pMG36e-S1 and MG1363/pMG36e-M) via oral administration in BALB/c mice and neonatal piglets, assessing cellular, humoral, and mucosal immune responses in the host. The results demonstrated that the recombinant strains significantly stimulated lymphocyte proliferation in mice and increased the proportion of CD3+, CD4+, and CD3+, CD8+ double-positive cells in the spleens of mice and the peripheral blood of piglets (*p* < 0.05). Furthermore, the recombinant strains significantly increased serum IgG, IgA, and mucosal SIgA levels in piglets (*p* < 0.05). Meanwhile, serum cytokine levels, including IL-4 and IFN-γ, were significantly elevated in piglets when compared to the control group (*p* < 0.05). In conclusion, the recombinant *L. lactis* demonstrated promising potential as a novel live vector vaccine against PEDV.

## 1. Introduction

Porcine epidemic diarrhea (PED) is a severe gastrointestinal disease caused by the porcine epidemic diarrhea virus (PEDV), which was first identified in the United Kingdom in the 1970s [1] and subsequently spread worldwide [2,3,4,5,6]. PEDV causes significant loss of appetite in pigs of all ages, accompanied by symptoms such as diarrhea and vomiting [7,8]. However, piglets are more susceptible to PEDV, with mortality rates reaching nearly 100% in severe cases, resulting in significant economic losses to the global swine industry [9,10].

PEDV is an enveloped virus containing a single-stranded, positive-sense RNA genome, classified in the genus Alphacoronavirus of the family Coronaviridae. The complete genome of PEDV is approximately 28 kilobases (kb) in length. The genome has a 5′ cap structure and a 3′ polyadenylation tail, and is structurally delineated into a 5′ untranslated region (UTR), a minimum of seven open reading frames (ORF1a, ORF1b, and ORFs 2–6), and a 3′ untranslated region [11,12]. ORF1a and ORF1b mainly encode replicase polyproteins, while ORF3 is involved in encoding non-structural proteins [13]. Furthermore, the remaining open reading frames encode the viral structural proteins, including the spike (S) protein, envelope (E) protein, membrane (M) protein, and nucleocapsid (N) protein [14,15]. Among these structural proteins, the S protein is a highly glycosylated type I membrane protein, with a molecular weight of 150–220 kDa, and plays a crucial role in inducing neutralizing antibody production. Interestingly, the S protein is divided into two subunits: S1 (1–789 aa) and S2 (790–1382 aa). The S1 subunit contains the main antigenic determinants and the receptor-binding domain (RBD), which is able to induce protective immunity in pigs [16]. Additionally, the M protein, a highly conserved multi-transmembrane protein on the viral envelope [17], plays a key role in viral assembly and budding, and induces protective antibodies with neutralizing activity. Therefore, the S1 subunit and M protein may serve as key targets for the development of effective PEDV vaccines.

Currently, vaccination is the primary method for controlling PED outbreaks. Various PEDV vaccines are commercially available, including inactivated and live attenuated vaccines [18]. Although these vaccines can provide a certain level of immune protection, limitations still exist. Inactivated vaccines have relatively low immunogenicity, often requiring multiple doses to achieve sufficient immune protection [19]. Additionally, they need to be administered with adjuvants to effectively induce mucosal immune responses [20], which further increases their cost. Furthermore, injectable immunization may induce stress responses in animals, whereas oral immunization offers advantages such as ease of administration, high compliance, and fewer adverse reactions, making it a milder vaccination approach [21]. Although oral live-attenuated vaccines can effectively stimulate the mucosal immune system, they may pose certain safety risks. Therefore, developing a novel vaccine which is safe, effective, and cost-efficient is crucial for preventing PEDV.

*Lactococcus lactis* (*L. lactis*) is a Gram-positive bacterium widely recognized as a “Generally Recognized as Safe (GRAS)” microorganism in the food industry [22]. *L. lactis* expression vectors are safe and non-toxic, free of endotoxins, and devoid of the safety risks associated with other live vaccine vectors [23]. The foreign proteins expressed by *L. lactis* can be orally administered with the bacterial cells, eliminating the need for purification. Additionally, *L. lactis*-based oral vaccines deliver antigens through the gastrointestinal mucosa, colonizing and persisting in the digestive tract, thereby mimicking the natural course of viral infection. This process effectively induces localized mucosal immune responses [24]. *L. lactis* is regarded as an ideal oral vaccine vector, demonstrating significant potential for future vaccine development.

Therefore, this study evaluated the immunogenicity of recombinant *L. lactis* expressing PEDV S1 and M proteins (MG1363/pMG36e-S1 and MG1363/pMG36e-M) in preventing PEDV, analyzing the immune responses of BALB/c mice and neonatal piglets by oral immunization, including cellular, humoral, and mucosal immunity. Our study provided a theoretical basis for the development of novel live vector-based oral vaccines for the prevention and control of PEDV.

## 2. Materials and Methods

### 2.1. Bacterial Strains

Recombinant *L. lactis* strains MG1363/pMG36e-S1 and MG1363/pMG36e-M were constructed and stored in our laboratory.

### 2.2. Experimental Animals

A total of 72 eight-week-old female BALB/c mice were procured from Jinan Pengyue Experimental Animal Breeding Co., Ltd. (Jinan, China) and underwent a 7-day acclimatization phase before the actual experiment. Throughout the duration of the experiment, they were housed under controlled conditions with a temperature of 23–25 °C, humidity of 40–60%, and a 12-h light/dark cycle. Simultaneously, they were allowed free access to food and water during both the acclimatization and experimental phases. A total of 21 healthy newborn crossbred piglets were supplied by Shandong Huahong Bioengineering Co., Ltd. (Binzhou, China). All of the piglets tested negative for PEDV, TGEV, PoRV, and PDCoV, as well as for PRRSV, PRV, and CSFV before the experiment. Throughout a 7-day acclimatization phase, all piglets were housed in a standardized facility at Shandong Huahong Bioengineering Co., Ltd., with free access to food and water according to institutional guidelines. After acclimating for 7 days, a 28-day experiment was conducted. All applicable international and national guidelines for the care and use of animals were followed. All of the animal experiments were approved by the Animal Ethics Committee of Qingdao Agricultural University with reference number QAU2024031102.

### 2.3. The Cultivation of Recombinant L. lactis

Recombinant *L. lactis* strains MG1363/pMG36e-S1 and MG1363/pMG36e-M were streaked onto GM17 agar plates and incubated anaerobically at 28 °C for 24 to 36 h. Single colonies were then aseptically picked and transferred to GM17 liquid medium for anaerobic incubation at 28 °C for 24 h. The culture was then inoculated into fresh GM17 liquid medium at a 2% ratio and incubated anaerobically at 28 °C for 48 h. The OD_600_ was measured at 0.8 to 1.0, corresponding to an approximate bacterial concentration of 1 × 10^8^ CFU/mL. The bacterial cells were harvested, washed with sterile PBS, and resuspended to a final concentration of 1 × 10^9^ CFU/mL.

### 2.4. Immunization Protocol for Mice

After acclimatization, eight-week-old female BALB/c mice were randomly assigned to two groups designated as the double immunization protocol (DI) and triple immunization protocol (TI) groups (n = 36 per group). Subsequently, mice from the DI and TI groups were divided into three subgroups (n = 12 per subgroup), respectively, based on recombinant *L. lactis* strains: CD, SD, and MD in the DI group and CT, ST, and MT within the TI group. The experiment lasted for 42 days, and both protocols used the same interval between each immunization, which was about 7 days. All immunizations were administered orally, and the mice were deprived of water and diet for 12 h before immunization. Immunization administration details for the DI and TI groups are presented in Table 1 and Table 2.

### 2.5. Immunization Protocol for Piglets

After a 7-day acclimatization, the piglets were randomly assigned to three groups (n  =  7 per group), which were named the C, S, and M groups. Piglets from the three groups were administered 3mL of PBS (C group), MG1363-pMG36e-S1 at 1 × 10^9^ CFU mL^−1^ (S group), or MG1363-pMG36e-M at 1 × 10^9^ CFU mL^−1^ (M group) via oral gavage, respectively. Also, these piglets were immunized twice with a 7-day interval between each immunization during the 28-day experiment. In other words, the piglets were immunized on day 0 and day 8. The specific administration program for the piglets is shown in Table 3.

### 2.6. Lymphocyte Proliferation Assay in Mice

Mice from the DI group on days 35 and 42 were euthanized, while mice from the TI group on day 42 were euthanized. Their spleens were harvested under aseptic conditions, placed on a 70 μm sterile cell sieve (Sangon Biotech (Shanghai) Co., Ltd., Shanghai, China), and ground into a cell suspension using a syringe plunger (Henan Shuguang Hzk Biological Technology Co., Ltd., Luohe, China). The lymphocytes on the cell sieve were rinsed with sterile PBS (Cytiva Bio-technology(Hangzhou) Co., Ltd., Hangzhou, China). The cell suspension was collected into a sterile centrifuge tube (Biosharp, Hefei, China), centrifuged at 2000 rpm for 10 min, and the supernatant was discarded. The cells were resuspended in red blood cell lysis buffer (Beijing Solarbio Science & Technology Co., Ltd., Beijing, China) and incubated for 10 min, after which the mixture was centrifuged at 2000 rpm for 10 min. After discarding the supernatant, the cells were rinsed twice with sterile PBS and once with RPMI-1640 medium. Finally, the cells were resuspended in RPMI-1640 medium (hyclone, Hangzhou, China) (containing 1% double antibiotics and 10% fetal bovine serum), and the concentration was adjusted to 1 × 10^7^ cells/mL to obtain the mouse spleen lymphocyte suspension. Five replicates were set up for each cell sample, with 100 µL of cell suspension added to each well of a 96-well plate. The 96-well plates (Beijing Labgic Technology Co., Ltd., Beijing, China) were incubated for 48 h at 37 °C with 5% CO_2_. Afterward, 10 µL of CCK-8 solution was added to each well, and the 96-well plates were incubated at 37 °C with 5% CO_2_ for 1 h. The absorbance at 450 nm was then measured using a microplate reader (Tecan, Männedorf, Switzerland).

### 2.7. Assay of Spleen Lymphocyte Subsets in Mice

The spleens of mice from the DI and TI groups were harvested under sterile conditions on day 35. A single-cell lymphocyte suspension was prepared using the same procedure as described in the lymphocyte proliferation assay. A 500 µL aliquot of each suspension was transferred into a sterile, enzyme-free 1.5 mL centrifuge tube (Biosharp, Hefei, China). Then, 2 µL of PE-CD3+, FITC-CD4+, and APC-CD8+ fluorescent antibodies (Miltenyi Biotec, Cologne, Germany) were added, mixed thoroughly, and incubated in the dark at 37 °C with 5% CO_2_ for 30 min. Subsequently, flow cytometry was used to detect CD3+ cells in the PE channel, CD4+ cells in the FITC channel, and CD8+ cells in the APC channel. Flow cytometry data were analyzed using FlowJo V10 software.

### 2.8. Assay of Peripheral Blood Lymphocyte Subsets in Piglets

On day 28, blood was collected from the jugular vein of piglets into EDTA anticoagulant tubes (Hebei Kangweishi Medical Technology Co., Ltd., Shijiazhuang, China). A 100 µL aliquot of anticoagulated blood was transferred into a 2 mL centrifuge tube (Biosharp, Hefei, China), and 2 µL of PE-CD3+, FITC-CD4+, and APC-CD8+ fluorescent antibodies were added and mixed. The tube was incubated at room temperature in the dark for 20 min. Then, 1.5 mL of red blood cell lysis buffer was added, and the sample was incubated in the dark for 10 min. The sample was centrifuged at 2000 rpm for 5 min, and the supernatant was discarded. The cells were resuspended in 1.5 mL of sterile PBS, mixed well, and centrifuged again at 2000 rpm for 5 min. The supernatant was discarded, and the cells were resuspended in 500 µL of sterile PBS. Finally, the samples were analyzed by flow cytometry. The method was similar to that previously mentioned in Section 2.7.

### 2.9. Assay of Cytokines IFN-γ and IL-4 in Piglets

Five piglets from each group were selected at different time points (days 0, 14, 21, and 28), and blood samples were collected from the jugular vein for serum isolation. The levels of IFN-γ and IL-4 in the serum samples were measured using the Pig Interferon-γ (IFN-γ) ELISA Kit (Wuhan Huamei Biological Engineering Co., Ltd., Wuhan, China) and the Pig Interleukin-4 (IL-4) ELISA Kit (Wuhan Huamei Biological Engineering Co., Ltd., Wuhan, China). Briefly, the negative control well contained 50 µL, the positive control well contained 50 µL, and the sample wells contained 10 µL of the sample to be tested and 40 µL of diluent. Each well was supplemented with 100 µL of HRP-labeled detection antigen, incubated at 37 °C for 1 h, then washed five times with wash buffer. Subsequently, each well was supplemented with 50 µL each of substrate A and substrate B and incubated for 15 min at 37 °C, avoiding light. Within 15 min, 50 µL of termination solution was added to each well, and after that, the absorbance was read at 450 nm using an enzyme-labeling measuring instrument.

### 2.10. Assay of Serum IgG Antibody in Piglets

Blood samples were collected from the jugular vein of piglets on days 0, 14, 21, and 28, and the serum was separated. The IgG levels in the serum samples were determined using the PEDV Antibody ELISA Kit (Shandong Lvdu Bio-sciences Technology Co., Ltd., Binzhou, China). The method was similar to that previously mentioned in Section 2.9.

### 2.11. Assay of Serum IgA Antibody in Piglets

On day 28, five piglets from each group were randomly selected, and blood samples were collected from the jugular vein for serum isolation. The IgA antibody levels in the serum samples were measured using the Pig IgA ELISA Kit (Abcam plc, Cambridge, UK). The method was similar to that previously mentioned in Section 2.9.

### 2.12. Assay of SIgA Antibodies in the Intestinal Mucosa and Feces of Piglets

Fourteen days after the second immunization, fecal samples were collected from piglets using a rectal swab method and placed into sterile centrifuge tubes. A volume of 2 mL of PBS was added to the centrifuge tube to immerse the rectal swab, followed by overnight incubation at 4 °C and subsequent centrifugation to obtain the fecal wash. The piglets were euthanized, and a 15 cm segment of their small intestine was excised. The intestinal mucosa was scraped into 2 mL of sterile PBS, mixed, and then centrifuged to obtain the intestinal mucosa wash. The SIgA antibody levels in both the fecal wash and intestinal mucosa wash were measured using the Pig IgA ELISA Kit (Abcam plc). The method was similar to that previously mentioned in Section 2.9.

### 2.13. Statistical Analysis

Data were analyzed using one-way analysis of variance (ANOVA) in IBM SPSS Statistics 25. A *p*-value of less than 0.05 was considered statistically significant, while a *p*-value greater than 0.05 indicated no significant difference.

## 3. Results

### 3.1. Results of Splenic Lymphocyte Proliferation Assay in Mice

**Figure 1:**
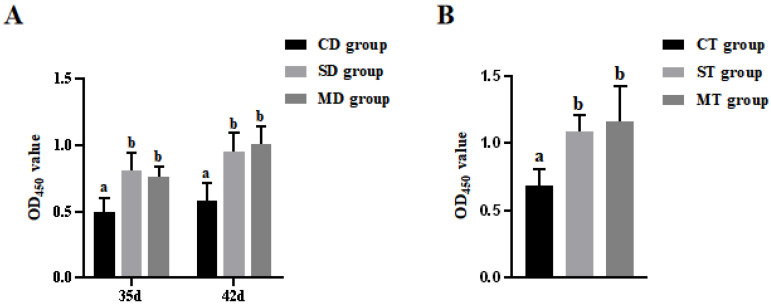
The levels of lymphocyte proliferation in mice. (**A**) The OD_450_ values in CD, SD, and MD groups. (**B**) The OD_450_ values in CT, ST, and MT groups. Different lowercase letters at the same time point indicate significant differences (*p* < 0.05).

Figure 1 shows the levels of lymphocyte proliferation in mice after oral immunization with recombinant *L. lactis*. With respect to the assessment of splenic lymphocyte proliferation in the DI group, significant differences were observed in both the SD and MD groups, in comparison to the CD group (*p* < 0.05); however, there were no significant differences between the SD and MD groups on days 35 and 42 (*p* > 0.05, Figure 1A). A similar trend in lymphocyte proliferation levels was observed in the TI group. To be specific, compared to the CT group, there were significant differences in the ST and MT groups (*p* < 0.05), while no statistically significant differences were seen between the ST and MT groups on day 42 (*p* > 0.05, Figure 1B). Notably, on day 42, the OD_450_ value of the ST group was slightly higher than that of the SD group, and the value of the MT group was slightly higher than that of the MD group; however, this tendency lacked statistical significance (*p* > 0.05).

### 3.2. Results of Splenic Lymphocyte Subset Assay in Mice

**Figure 2:**
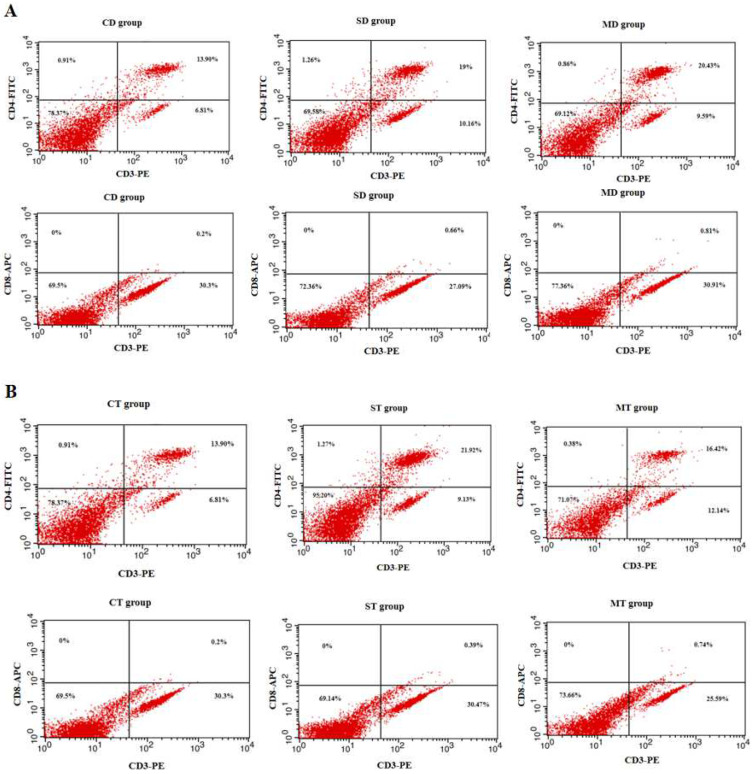
The changes in the number of CD3+, CD4+, and CD8+ cells in the mouse spleen. (**A**) The proportion of CD3+, CD4+, and CD3+, CD8+ double-positive cells in the CD, SD, and MD groups. (**B**) The proportion of CD3+, CD4+, and CD3+, CD8+ double-positive cells in the CT, ST, and MT groups.

The proportion of CD3+, CD4+, and CD8+ T cells in the spleens of each group was assessed by flow cytometry. On day 35, no significant changes in the proportion of CD3+, CD4+, and CD3+, CD8+ double-positive cells were found between the SD and MD groups (*p* > 0.05), but both groups exhibited significant differences compared to the CD group (*p* < 0.05, Figure 2A). In terms of the proportion of CD3+, CD4+, and CD3+, CD8+ double-positive cells in the TI group, an identical trend was seen, with significantly higher numbers in both the ST group and MT group in comparison to the CT group (*p* < 0.05), while no statistical significance was observed between ST and MT groups (*p* > 0.05, Figure 2B). The proportion of double-positive cells in the ST group was slightly higher than that in the SD group, and the proportion of double-positive cells in the MT group was slightly higher than that in the MD group, though the difference was not significant (*p* > 0.05).

### 3.3. Results of Peripheral Blood Lymphocyte Subset Assay in Piglets

**Figure 3:**
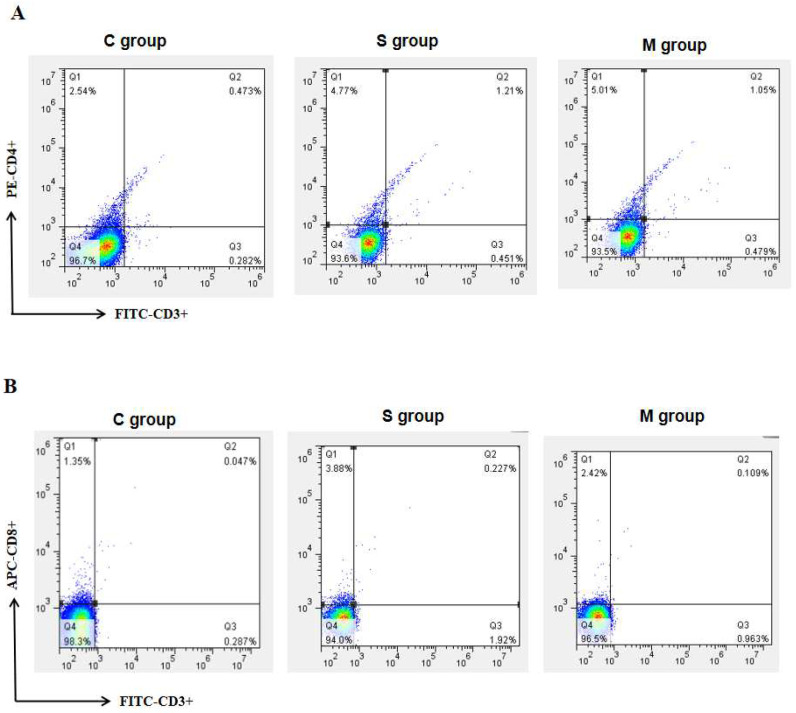
The changes in the number of CD3+, CD4+ (**A**) and CD3+, CD8+ (**B**) double-positive cells in piglet peripheral blood.

Blood lymphocyte subsets in newborn piglets from each group were analyzed by flow cytometry. For CD3+, CD4+ double-positive cells, compared to the C group, the proportion was significantly increased in both the S and M groups on day 28 (*p* < 0.05, Figure 3A). Similarly, a significant increase in the number of CD3+, CD8+ double-positive cells in the S and M groups was observed on day 28 (*p* < 0.05, Figure 3B).

### 3.4. Results of Cytokines Assay in Piglets

**Figure 4:**
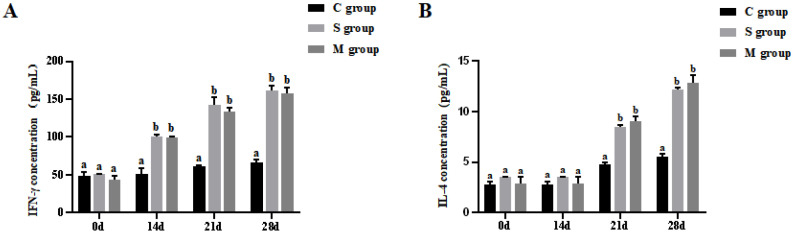
The levels of cytokines IFN-γ (**A**) and IL-4 (**B**) in piglets at different time points. Different lowercase letters at the same time point indicate significant differences (*p* < 0.05).

After oral immunization with recombinant *L. lactis*, the levels of IFN-γ and IL-4 were significantly elevated in the serum of piglets from both the S and M groups compared to the C group (*p* < 0.05, Figure 4A,B). However, no noteworthy difference existed between the S and M groups (*p* > 0.05). With respect to the changes in both of the cytokines during the experiment, there was an observable increase in each group from day 0 to day 28.

### 3.5. Results of Serum IgG Antibody Assay in Piglets

**Figure 5:**
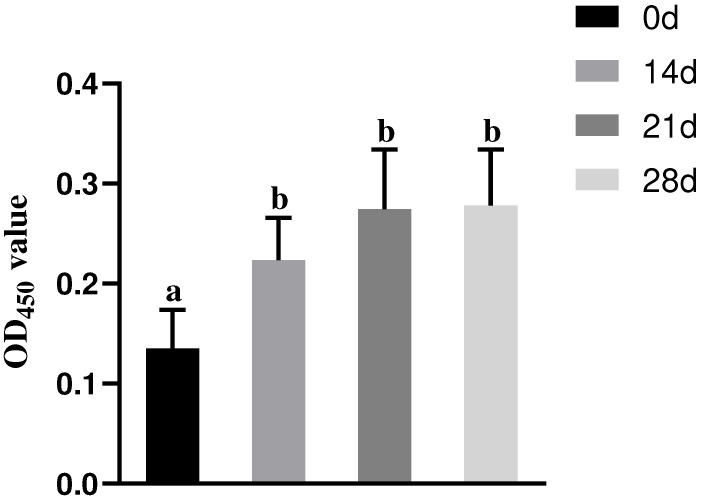
The changes in serum IgG antibody levels of piglets in the S group over time. Different lowercase letters indicate significant differences (*p* < 0.05) between groups.

IgG levels in the serum of piglets were measured by ELISA. During the 28-day experiment, IgG antibody levels gradually increased over time in the S group, with the IgG levels in the serum samples on days 14, 21, and 28 being significantly higher than those on day 0 (*p* < 0.05, Figure 5).

### 3.6. Results of Serum IgA Antibody Assay in Piglets

**Figure 6:**
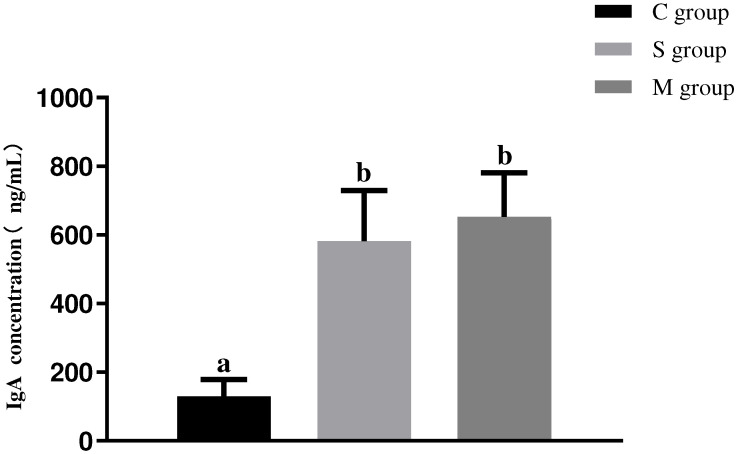
The serum IgA antibody level in piglets. Different lowercase letters indicate significant differences (*p* < 0.05) between groups.

As shown in Figure 6, the results indicated that oral administration of recombinant *L. lactis* significantly increased serum IgA antibody levels. Specifically, the serum IgA levels in both the S and M groups were significantly higher than those in the C group (*p* < 0.05). However, no significant difference was observed in serum IgA antibody levels between the S and M groups (*p* > 0.05).

### 3.7. Results of SIgA Antibody Assay in Intestinal Mucosa and Feces of Piglets

**Figure 7:**
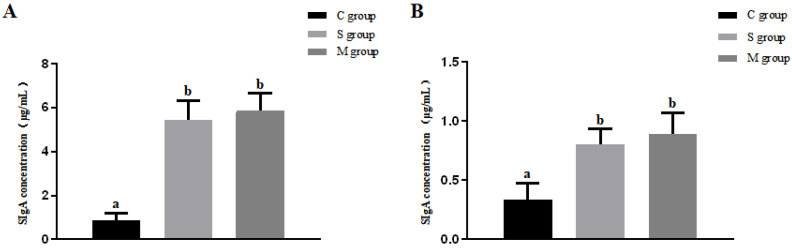
Intestinal mucosal (**A**) and fecal (**B**) SIgA antibody levels in piglets. Different lowercase letters indicate significant differences (*p* < 0.05) between groups.

Mucosal and fecal wash samples were collected from piglets on day 28, and SIgA antibody levels were measured by ELISA. As shown in Figure 7, despite showing no significant difference between the S and M groups (*p* > 0.05), oral administration of recombinant *L. lactis* significantly increased SIgA antibody levels in both the mucosal and fecal washes in these two experimental groups compared to the C group (*p* < 0.05).

## 4. Discussion

Continuous mutations of PEDV have led to the emergence of various strains [25]. Currently, researchers classify these strains into two genotypes: GI and GII [12]. In 2010, a highly virulent GII variant strain emerged, leading to a large-scale outbreak of PED worldwide [26,27] and causing significant economic losses to the global swine industry. Traditional vaccines are less effective in preventing infection by these variant strains. Consequently, researchers have developed new inactivated and attenuated live vaccines targeting the GII variant to control and prevent PEDV infections [28,29]. Although these vaccines have demonstrated strong protective effects, they still have certain limitations. For instance, inactivated vaccines are constrained by their short-lived immunity and the necessity for high immunization doses. Additionally, their primary mode of administration is via injection, which often requires adjuvants to effectively induce mucosal immune responses [20], thereby increasing overall costs. Although oral live-attenuated vaccines effectively stimulate the mucosal immune system, they pose challenges in terms of storage and transportation and carry the risk of virulence reversion. Consequently, there is an urgent need to develop a novel PEDV vaccine that integrates efficient mucosal immune activation, safety, and cost-effectiveness. This study utilized recombinant *L. lactis* for oral immunization of mice and neonatal piglets, inducing robust mucosal and systemic immune responses. These findings provide a foundation for the development of effective oral vaccines against PEDV infection.

*L. lactis* is a microorganism widely distributed in nature, commonly found in the gastrointestinal tracts of various animals [30]. It demonstrated a high survival rate in the digestive tract, exhibiting remarkable tolerance to the harsh conditions of the gastrointestinal environment. *L. lactis* can colonize the intestinal tract, effectively stimulating immune responses and promoting immune tolerance in the host [31]. The peptidoglycan in its cell wall acts as an immunoadjuvant, further enhancing the immune response [32]. Notably, *L. lactis* is considered a safe microorganism, since it is non-toxic and free from adverse side effects. Recombinant strains of *L. lactis* can be administered orally, inducing robust immune responses in the host. Given these advantages, researchers generally consider *L. lactis* an ideal host for the efficient expression of foreign proteins. Gao et al. (2015) constructed a recombinant *L. lactis* expressing the ORF2 gene of Hepatitis E virus (HEV). Oral immunization of mice with this strain elicited a robust mucosal and systemic immune response [33]. Wang et al. (2019) constructed a recombinant *L. lactis* oral vaccine expressing a fusion protein of RCK and IBDV VP2. Animal experiments demonstrated that the vaccine could induce high levels of neutralizing antibodies and effectively protect chickens from IBDV infection [34]. Wang et al. (2013) constructed a recombinant *L. lactis* live vector vaccine based on the ORF6 gene of Porcine Reproductive and Respiratory Syndrome (PRRS). The results indicated that the recombinant strain could induce significant mucosal immunity, humoral immunity, and Th1-type cellular immunity in mice [35]. Therefore, using *L. lactis* as an expression vector for the preparation of oral live vector vaccines offers significant advantages.

Cellular immunity plays a crucial role in combating infections caused by foreign pathogens. CD3 is a key surface marker of T lymphocytes. T lymphocytes are further classified into helper T cells (CD4+ T cells) and cytotoxic T cells (CD8+ T cells) based on their surface markers. During pathogen invasion, activated CD4+ and CD8+ T cells recognize antigens presented by antigen-presenting cells and release a variety of cytokines to mediate immune responses [36]. In this experiment, recombinant *L. lactis* was used for oral immunization of mice, with the immunization protocols consisting of either a two-dose or a three-dose regimen. The changes in the numbers of splenic CD3+, CD4+, and CD8+ cells in each group of mice on day 35 were analyzed by flow cytometry. The results showed a significant increase in the proportion of CD3+, CD4+ and CD3+, CD8+ cells in experimental groups (*p* < 0.05). However, no significant difference was observed between the experimental groups (*p* > 0.05). The proportion of double-positive cells in the ST and MT groups on day 42 was slightly higher than that in the SD and MD groups on day 42, but the difference was not statistically significant (*p* > 0.05). This indicates that recombinant bacteria can effectively stimulate T cell maturation and induce an effective cellular immune response in the host.

Lymphocyte proliferation levels serve as indicators of lymphocyte activity and functionality. Previous studies have shown a positive correlation between lymphocyte proliferation levels and cellular immune responses [37]. In our study, the proliferative activity of splenic lymphocytes was assessed. The results showed that the proliferative activity of splenic lymphocytes in the experimental groups was significantly higher than that in the control group (*p* < 0.05), while no significant difference was observed between the experimental groups (*p* > 0.05). The lymphocyte proliferation activity of the ST and MT groups on day 42 was slightly higher than that of the SD and MD groups on day 42, but the difference was not statistically significant (*p* > 0.05). This indicated that the recombinant bacteria can induce a robust cellular immune response in mice. Lin et al. (2022) used recombinant *Bacillus subtilis* (*B. subtilis*) for oral immunization of mice, inducing a robust cellular immune response [38], which is consistent with the findings of this study. Additionally, the results of this study suggest that the two-dose immunization regimen can achieve immune effects comparable to those of the three-dose regimen. This may be attributed to the fact that, by the second immunization, memory lymphocytes are already sufficiently developed to provide an adequate level of protection. Over time, the immune response in animals may reach a saturation point, beyond which increasing the number of immunizations may not significantly enhance the immune response. Piglets are the natural hosts of PEDV. Building on the mouse model, we proceeded to use recombinant *L. lactis* for oral immunization of neonatal piglets. On day 28, blood was collected from the jugular vein, and lymphocytes were subsequently isolated. Flow cytometry was then employed to analyze and detect the cells. The results demonstrated that the recombinant bacteria significantly increased the proportion of CD3+, CD4+, and CD3+, CD8+ double-positive lymphocytes in the peripheral blood of piglets. These findings suggested that recombinant *L. lactis* can induce cellular immune responses in piglets.

In the adaptive immune response, helper T cells (Th) are an essential subpopulation of the immune system. They play a crucial role in producing cytokines and assisting other immune cells in executing specific functions. Among them, Th1 cells primarily secrete cytokines such as IFN-γ and IL-2, which promote cell-mediated immune responses, while Th2 cells primarily secrete cytokines such as IL-4 and IL-6, which mediate humoral immunity [39,40]. The level of cellular immunity in the organism can be partially assessed by changes in the cytokine levels of IL-4 and IFN-γ. In this study, recombinant Lactobacillus strains were orally administered to neonatal piglets, and the serum cytokine levels of IL-4 and IFN-γ were measured by ELISA. The results showed that the levels of IL-4 and IFN-γ in the experimental groups were significantly higher than those in the control group (*p* < 0.05). It suggested that the recombinant bacteria can induce effective Th1 and Th2 type cellular immune responses in the organism, consistent with the findings of Wang et al. (2017) [41].

Oral immunization is an effective strategy for preventing enteric infectious diseases. This vaccination method is easy to administer, highly safe, and capable of directly blocking pathogens from entering the mucosal surfaces of the digestive tract. Its superiority over other immunization routes lies in its capacity to stimulate local immune cells in the gut to produce SIgA, thereby inducing systemic immunity [42]. The concentration of SIgA in the intestinal mucosa is significantly higher than that of other immunoglobulins, making SIgA crucial for intestinal mucosal immunity [43]. SIgA prevents pathogens and toxins from adhering to the intestinal mucosa, thereby protecting it from damage and inhibiting the widespread invasion of pathogens [44,45]. Studies have shown that, for many viruses, including respiratory and gastrointestinal viruses, oral immunization is more effective than conventional vaccination, as it induces SIgA and IgG antibodies while stimulating humoral, cellular, and mucosal immune responses [46]. Additionally, oral vaccines have been shown to increase serum levels of IgA [47,48]. Previous studies have shown that the neutralizing activity against PEDV is significantly correlated with IgA levels in colostrum [49]. Furthermore, serum IgA levels are strongly correlated with colostrum IgA, suggesting that serum IgA could serve as a viable alternative indicator for evaluating IgA levels in colostrum samples [50]. Additionally, Santaolalla et al. (2021) also demonstrated that serum IgA levels were an important indicator for evaluating humoral immune status [51]. Therefore, these findings suggested that serum IgA antibodies play an important role as an indicator for evaluating the immune protective efficacy of oral vaccines. In this study, piglets were orally immunized with recombinant *L. lactis*, and the results showed that the recombinant bacteria significantly increased the levels of mucosal SIgA, serum IgG, and serum IgA antibodies in the piglets (*p* < 0.05). Wang et al. (2019) orally immunized piglets with recombinant *B. subtilis*, inducing significant mucosal and humoral immune responses, which is consistent with the findings of this study [52]. These findings suggest that the recombinant bacteria are capable of inducing effective mucosal and humoral immunity in piglets. However, the long-term immune protection conferred by the immunization of recombinant bacteria still requires further evaluation.

While this study has demonstrated that recombinant *L. lactis* exhibits significant immunogenicity in both mouse and piglet models, the highly controlled laboratory environment cannot fully replicate the complex conditions encountered in commercial pig herds under actual production settings. Factors such as temperature variations, hygiene standards, and co-infections may significantly influence the immunological efficacy of oral vaccines. To comprehensively evaluate the practical application potential of this vaccine, further studies are essential to assess its immunogenicity and protective efficacy under real-world production conditions. Additionally, the genetic diversity of PEDV presents a considerable challenge to vaccine-induced protection, underscoring the necessity for developing multivalent vaccines capable of providing broad-spectrum protection, which should be a key focus of future research efforts.

## 5. Conclusions

Recombinant *L. lactis* MG1363/pMG36e-S1 and MG1363/pMG36e-M exhibited robust immunogenicity, effectively inducing cellular, humoral, and mucosal immune responses. These findings suggest that these recombinant strains hold significant promise as novel oral vaccine candidates for the prevention and control of PEDV, thereby addressing the limitations of traditional vaccines.

## Figures and Tables

**Table 1:** Administration program for the mice in DI group.

Group	Recombinant *L. lactis* Strains	Dose	Number of Immunizations
CD	-	300 µL of PBS	2
SD	MG1363-pMG36e-S1	300 µL of 1 × 10^9^ CFU mL^−1^	2
MD	MG1363-pMG36e-M	300 µL of 1 × 10^9^ CFU mL^−1^	2

**Table 2:** Administration program for the mice in TI group.

Group	Recombinant *L. lactis* Strains	Dose	Number of Immunizations
CT	-	300 µL of PBS	3
ST	MG1363-pMG36e-S1	300 µL of 1 × 10^9^ CFU mL^−1^	3
MT	MG1363-pMG36e-M	300 µL of 1 × 10^9^ CFU mL^−1^	3

**Table 3:** Administration program for the piglets.

Group	Recombinant *L. lactis* Strains	Dose	Number of Immunizations
C	-	3 mL of PBS	2
S	MG1363-pMG36e-S1	3 mL of 1 × 10^9^ CFU mL^−1^	2
M	MG1363-pMG36e-M	3 mL of 1 × 10^9^ CFU mL^−1^	2

## Data Availability

The original contributions presented in the study are included in the article. Further inquiries can be directed to the corresponding authors.
